# A novel device for assessing dark adaptation in field settings

**DOI:** 10.1186/s12886-015-0062-7

**Published:** 2015-07-09

**Authors:** Alain B. Labrique, Amanda C. Palmer, Katherine Healy, Sucheta Mehra, Theodor C. Sauer, Keith P. West, Alfred Sommer

**Affiliations:** Department of International Health, Johns Hopkins Bloomberg School of Public Health, Baltimore, MD USA

**Keywords:** Dark adaptometry, Pupil dynamics, Pupillary threshold, Night blindness, Vitamin A

## Abstract

**Background:**

Aberrant dark adaptation is common to many ocular diseases and pathophysiological conditions, including vitamin A deficiency, cardiopulmonary diseases, and hypoxia. Scotopic vision and pupillary responsiveness have typically been measured using subjective, time-consuming methods. Existing techniques are particularly challenging for use in developing country settings, where vitamin A deficiency remains a major public health problem. Our aim was design a compact, low cost, and easily operated device to assess dark adaptation in the field.

**Methods:**

The Portable Field Dark Adaptometer (PFDA) incorporates a digital camera, a retinal bleaching flash, and a Ganzfeld light source inside a pair of light-obscuring goggles. After a ~10 min period of dark adaption, the infrared camera digitally records afferent pupillary responses to graded light stimuli (−2.9 to 0.1 log cd/m^2^). We tested this device in a variety of field settings to assess: a) ease of use and b) whether test data could clearly and accurately depict the well-known dose-response relationship between light intensity and pupil contraction. A total of 822 videos were collected. We used an open source video analysis software to measure pupil size in pixel units. Pupillary responsiveness was expressed as the percent change in pupil size from pre- to post-light exposure. Box plots, t test, and multi-level mixed effects linear regression modeling were used to characterize the relationship between light intensity and pupillary response.

**Results:**

The PFDA was employed with only minor technical challenges in Bangladesh, Kenya, Zambia, and Peru. Our data show a clear linear increase in pupillary constriction with increasing log light intensity. Light intensity was a strong predictor of pupillary response, regardless of baseline pupil size.

**Conclusions:**

The consistent physiological response demonstrated here supports the use of the PFDA as a reliable tool to measure dark adaptation. As a next step, PFDA measurements will be validated against biochemical indicators of vitamin A status and hypoxemia. Ultimately, this new technology may provide a novel approach for nutritional assessment, with potential clinical applications.

**Electronic supplementary material:**

The online version of this article (doi:10.1186/s12886-015-0062-7) contains supplementary material, which is available to authorized users.

## Background

Dark adaptation is the visual adjustment that occurs when transitioning from a high- to low-light settings. It is characterized by pupil dilation and increased activity of the rod photoreceptors that line the retina. A wide variety of pathophysiological conditions can impair dark adaptation. The biochemical relationship between vitamin A and scotopic vision was first described in 1925 when Holm observed that regeneration of visual purple (rhodopsin) was slowed in vitamin A deficient rats [1]. The eye’s adaptability in dim light is also compromised in hypoxic individuals. This was first noted in fighter pilots during World War II, who described difficulties with their vision when flying at high altitudes; mountain climbers are similarly affected [2]. There is now a well-documented association between oxygen deficiency and impaired dark adaptation [3]. Night vision is extremely sensitive to even mild hypoxia, regardless of the underlying cause. For example, dark adaptation is impaired in patients with carotid artery disease due to their decreased arterial oxygen saturation [4].

Given the functional consequences of impaired dark adaptation, it has been extensively studied in both academic and military research as a sequelae of disease and marker of individual capacity [5, 6]. Tests for dark adaptation have typically focused on identification of a patient’s pupillary threshold using a stepwise series of light intensities [7]. The pupillary threshold is the lowest light intensity to cause a significant pupillary contraction in a dark-adapted eye [8]. Over a dozen dark adaptometers were produced during the 1940s, ranging from Wald’s “portable dark adaptometer” for vitamin A research to the British Army Medical Service’s “Middle East adaptometer” designed to test night vision in soldiers [1, 9–11]. These devices relied on subjective responses by an examiner and, in the end, did not correlate with biochemical measures of vitamin A status [12]. Until recently, the Goldmann-Weekers dark adaptometer (Haag-Streit) served as the gold-standard for to assess dark adaption; however, its size and complexity rendered this device unsuitable for field use.

To address the need for a portable and relatively inexpensive calibrated light source, Congdon *et al* developed and tested the Scotopic Sensitivity Tester-1 (SST-1; LKC Technologies, Maryland, USA). They found that SST-1 measures were significantly correlated with serum retinol concentration [8, 13]. In the hands of trained personnel, the SST-1 showed potential as a non-invasive measure of population vitamin A status [14, 15]; however, it still relied on subjective measurements, as an examiner had to observe “major” pupillary changes, and required a dark room. Nearly a century after the first proposal that dark adaptation could serve as an indirect measure of vitamin A status, we remain without a reliable, field-friendly dark adaptometer.

We tested the ease of use and performance of a novel Portable Field Dark Adaptometer (PFDA) under field conditions. Performance was graded on the device’s ability to accurately and clearly depict the well-known dose-response relationship between pupil contraction and light intensity.

## Methods

### Device design and testing

**Fig. 1:**
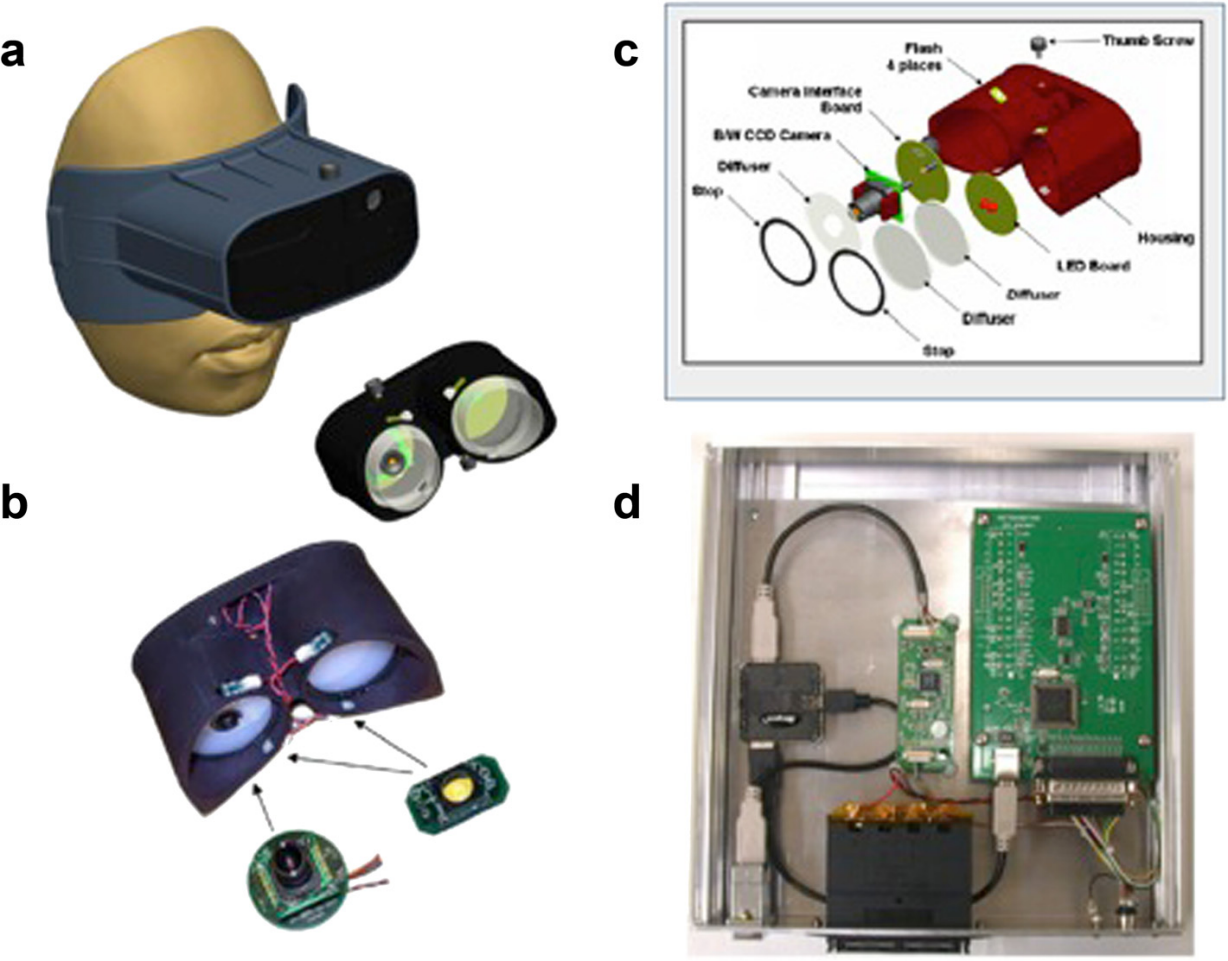
Schematic diagram of the Portable Field Dark Adaptometer (PFDA) developed to assess impaired pupillary responses to a graded series of Ganzfeld light stimuli applied within a pair of “dark-room” goggles (**a,b**) with an embedded microcircuit design (**c**) and regulated by a laptop-powered controller box (**d**)

**Fig. 2:**
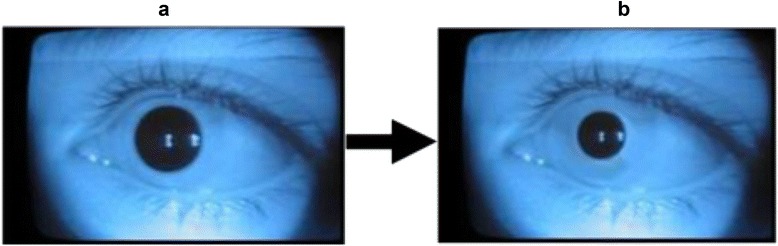
Infrared imaging allows the visualization of an eye in complete “dark-room” conditions while also enhancing pre (**a**) and post-stimulus (**b**) pupillary measurement due to the clear differentiation of the pupil-iris boundary, irrespective of iris color

The PFDA is comprised of a rubber shell housing, designed to fit snugly to the contours of the face, with soft foam padding around the eyes to ensure that no light enters once the goggles are in place. It is secured with a wide head strap (Fig. 1). This design creates a mobile dark room such that assessments can be done outdoors or in ambient light settings. An infrared mini-camera is mounted inside the right eyepiece to record pupillary response and aberrant activities (e.g., blinking, looking away) that may impact data quality. The infrared technology enables examiners and readers to easily distinguish between the pupil and iris, regardless of the color of the iris (Fig. 2). Testing stimuli are generated from a Ganzfeld (whole retina) 526 nm wavelength (green) light emitting diode (LED). Both eyepieces contain discrete LED flash units for baseline retinal bleaching. For test stimuli in the left eye, the pattern can be customized from a continuous linear trajectory to a stepwise series of 0.4 log increments over 12 steps, spanning −3.57 to 0.348 log cd/m^2^. Retinal bleaching and light intensity, sequence, and stimulus timing are all controlled by custom software on a laptop or netbook. The software interface records and links patient information to the PFDA test, prompts and guides examiners through the evaluation procedures, and stores and saves the video files for later quality control and analysis. Other components used in the assembly of the PFDA were modular and off-the-shelf to permit inexpensive manufacturing as well as replacement or service when needed.

To test the quality of the Ganzfeld illumination, a comparison of the angular profile (dropoff) in light intensity was performed on both the SST-1 device and a mock-up of the dual diffuser stack as constructed in the PFDA. The measurement was made by placing a silicon photodiode 2.75" in front of the dual diffuser stack (or the SST-1/VA-20 housing) while rotating the device through a range of −70 to +70°.

### Field testing

We developed our first iteration of the PFDA in 2003 during the initial phases of the JiVitA-1 vitamin A supplementation trial in Bangladesh [16]. Our aim in testing the first prototype was to determine the minimum time necessary for the dark adaptation process to be fully completed after retinal bleaching (data unpublished). With the development of new prototypes came field acceptability studies in Kenya and Bangladesh among married women of reproductive age and school-aged children, respectively (Table 1). Technical specifications in this manuscript refer to the currently available PFDA model. This standardized, field-ready, and precisely calibrated prototype was tested in a sample of preschool-aged children in Zambia and in Peruvian adults. Bangladesh, Kenya, and Zambia were targeted due to their classification as countries with a severe vitamin A deficiency public health problem, i.e., where ≥20 % of preschool-aged children or pregnant women [17]. PFDA assessments were added to ongoing research in all three countries to test the impact of vitamin A deficiency control interventions. The research site in Peru was established to study geographic variation in chronic disease risk [18]. PFDA measurements were added based on the hypothesis that, in individuals with low arterial oxygen saturation, there would be inadequate provision of oxygen to the retina, negatively impacting dark adaptation. Details of these study populations and PFDA summary measurements are presented in Table 1. Study protocols were approved by Institutional Review Boards at the Johns Hopkins Bloomberg School of Public Health (Bangladesh, Zambia, and Peru) and Wageningen University (Kenya), as well as by local ethics review committees (the Bangladesh Medical Research Council; the Ethical Review Board of the Tropical Diseases Research Centre in Zambia; the Institutional Review Board at Universidad Peruana Cayetano Heredia in Peru, and the Ethics and Research Committee of Kenyatta National Hospital in Kenya). Written (Peru and Kenya) or oral consent (Bangladesh and Zambia) were obtained from either study subjects (Bangladesh and Peru) or their parents (Kenya and Zambia).

**Table 1:** Pupil response metrics in four study populations using the Portable Field Dark Adaptometer (2010–2013)

Country, Assessment Year	Bangladesh, 2010	Kenya, 2010	Zambia, 2012	Peru, 2013
Study Population	Pregnant women	School-aged children	Preschool-aged children	Adults
n	242	184	305	91
Male, *n (%)*	0 (100.0)	79 (43.0)	148 (48.5)	44 (48.4)
Mean age ± SD	23.4 ± 6.1	9.2 ± 1.9	5.7 ± 1.3	55.1 ± 10.9
Mean relative change ± SD in pupil diameter (%)^1^				
All stimuli: -2.9 to 0.1 log cd/m^2^	−19.8 ± 5.0	−15.5 ± 5.8	−17.0 ± 6.8	−22.7 ± 8.0
Low intensity: −2.9 to −1.3 log cd/m^2^	−14.5 ± 5.3	−9.4 ± 5.1	−9.2 ± 5.9	−16.3 ± 7.6
High intensity: −0.9 to 0.1 log cd/m^2^	−26.2 ± 5.4	−23.1 ± 7.5	−26.7 ± 8.3	−30.5 ± 8.8
Mean response time (s) ^2^				
All stimuli: −2.9 to 0.1 log cd/m^2^	1.27 ± 0.31	1.02 ± 0.16	1.11 ± 0.21	1.13 ± 0.18
Low intensity: −2.9 to −1.3 log cd/m^2^	1.21 ± 0.29	0.90 ± 0.19	1.00 ± 0.21	1.07 ± 0.22
High intensity: −0.9 to 0.1 log cd/m^2^	1.35 ± 0.44	1.17 ± 0.17	1.24 ± 0.25	1.20 ± 0.22
Mean +/- SD pupillary threshold (log cd/m^2^)	−1.87 ± 0.75	−1.20 ± 0.83	−1.34 ± 0.70	−1.91 ± 0.81
Distribution of pupillary thresholds (%)^3^				
Good: −2.9 to −2.1 cd/m^2^	49.2	23.4	20.7	59.3
Adequate: −1.7 to −0.9 cd/m^2^	42.1	42.9	55.3	29.7
Impaired: −0.5 to 0.1 cd/m^2^	8.7	33.7	24.0	11.0

^1^Pre- to post-stimulus change pupil diameter (in pixels), expressed as proportion of pre-stimulus diameter; figures are mean +/- SD; lower values reflect a greater response/better dark adaptation

^2^Absolute value of the difference in video frame number from pre- to post-stimulus, divided by 30 frames/s; higher values reflect a faster response/better dark adaptation

^3^Pupillary threshold defined as first stimulus at which pupil diameter decreased by 20 % or more; abnormal as defined by Congdon et al. [8], i.e., pupillary threshold > = −0.5 log cd/m^2^

Training requirements for operation of the PFDA are minimal. In each of the sites, examiners were trained in a half-day session by one of the device’s developers (ABL). Training included an overview of dark adaptation, introduction to the device, use of the custom software, interactions with study subjects, testing procedures (described below), and trouble-shooting in the field. All trainees tested the PFDA procedures on volunteers. The strongest candidates were selected based on the developer’s assessment of aptitude, with emphasis on the trainee’s ability to interact and guide subjects through the testing phase. Selected examiners continued with practice-testing on a daily basis for approximately one week, with oversight and regular feedback provided by a supervisor.

### Assessment procedure

Examiners first attach the PFDA googles and adjust them until the subject is comfortable. He or she checks the perimeter of the goggles using a bright flashlight to identify and correct errant light penetration through gaps. After instructing the subject to open his or her eyes and look forward, the examiner initiates the test using the custom software. This begins with the bleaching of both retinas with a bright flash of light, exceeding 3400 cd-s/m^2^. A 10-min countdown then ensues, during which time the subject’s vision is expected to fully dark-adapt. The 10-min adaptation period is based on previous studies of dark adaptation, as well as testing results from the first PFDA prototype [8, 15]. A set of toy plastic animals was used during the dark adaptation period of younger children, to keep them from touching their goggles and disturbing their dark adaptation process. They were asked to feel and guess the animal as a game. At the end of the adaptation period, the software issues a warning to the operator that the test phase is about to begin. He or she then directs the subject to open their eyes and look forward. The exam protocol consists of nine light stimuli ranging from −2.9 to 0.1 log cd/m^2^, comparable to previous studies using the SST-1 device [8, 15]. Stimuli increase in increments of 0.4 log cd/m^2^ and last for one second each. There is a 10-s rest between stimuli to provide the pupil time to re-dilate to its pre-stimulus size. Prior to each of the nine stimuli, participants are reminded to open their eyes wide and look straight forward. They are also asked to blink as little as possible during the test. Aberrant activities like blinking and looking away are also recorded and can be actively monitored and discouraged during the testing window. At the end of test, the video is automatically saved on the laptop or netbook. Overall, the duration of PFDA testing procedures is approximately 15 min per subject.

### Video analysis

PFDA video recordings are assessed by a trained reader using Tracker 4.85 (Douglas Brown, Davidson, North Carolina), an open source video analysis and modeling software. The software’s “Tape Measure” tool enables readers to measure pupil diameter in pixels for any selected frame. For each subject’s video, data are recorded on the pupil’s starting diameter. The PFDA software adds an “LED” caption in the upper left-hand corner of all video frames recorded during each one-second stimulus. Pre-stimulus diameter is measured one frame prior to light exposure. The post-stimulus measurement is taken at the frame showing the smallest pupil diameter (Fig. 4). In addition to the pupil measurements, the reader records pre- and post-stimulus frame numbers. These can be used to calculate pupil dynamics such as velocity of response and pupil resent time. Additionally, quality assurance indicators are recorded for each light stimulus and/or the test as a whole, including: blinking; pupil not centered in image; presence of fog on the lens; and light let into the goggle, e.g., if the goggles were adjusted. This information can also be used in assessing data quality, rating testers, and providing feedback to the field. Video files from children with adequate [see Additional file 1] and impaired dark adaptation [see Additional file 2] are available as online supplementary materials.

### Data analysis

Three primary analytic metrics were developed for the PFDA: pupillary responsiveness (R), dynamics (D), and threshold (PT). Pupillary responsiveness is calculated as the percent change in pupil diameter from pre- to post-stimulus. Subjects with better dark adaptation would have a stronger, i.e., a more negative, response. Pupil dynamics are calculated by taking the absolute value of the difference in frame numbers from pre- to post-stimulus and then dividing by 30, i.e., the number of frames per second. A faster response would be expected of healthy subjects compared to impaired ones. Each subject’s test yields nine pupillary response variables and nine variables related to pupil dynamics. For analysis purposes, these variables are generally summarized by taking the mean responsiveness or mean time over all nine stimuli (−2.9 to 0.1 log cd/m^2^), low light stimuli (−2.9 to −1.3 log cd/m^2^), or high light stimuli (−0.9 to 0.1 log cd/m^2^). The latter grouping, referred to here as “high light stimuli,” is intended to capture the range of stimuli where even a vitamin A deficient population would be expected to respond [7]. Pupillary threshold is defined as the lowest light intensity that stimulated a ≥15 % relative change in the subject’s pupil diameter. Pupillary threshold, when evaluated by Congdon et al, was originally coded based on the stimulus number (i.e., stimulus 1 through stimulus 9). These were converted to light intensity (log cd/m^2^), based on the device’s initial calibration. Lower pupillary threshold values have historically been associated with healthy, vitamin A replete individuals, interpreted as a lower intensity of light required to trigger a significant, or major, response. Abnormal or “impaired” dark adaptation has been defined by a pupillary threshold ≥ -0.5 log cd/m^2^. This cut-off was based on data from 56 healthy American children with dark irises and has been employed previously in research using the SST-1 device [8, 14]. For analysis, we used box plots to examine distributions of pupillary response to individual light stimuli. A multilevel mixed effects model with undefined covariance was used to test the effect of light intensity on pupillary responsiveness, as characterized by the PFDA, controlling for starting pupil diameter. All statistical analyses were performed with Stata 12 statistical software (StataCorp LP, College Station, Texas).

## Results and discussion

**Fig. 3:**
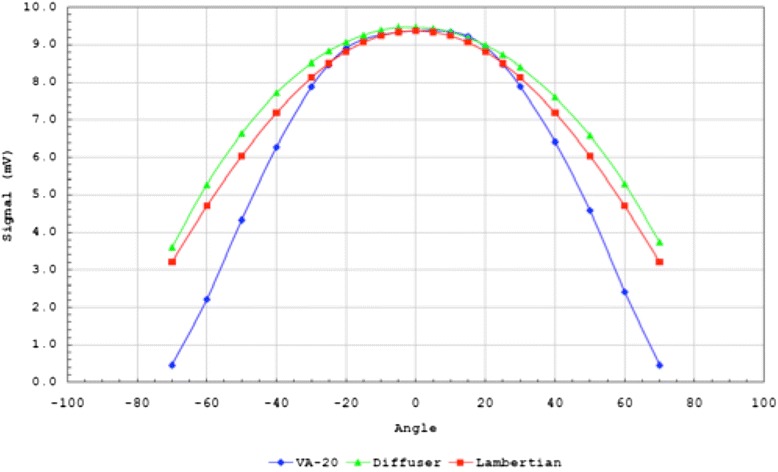
Comparison of the angular profile in light intensity between the VA-20 Ganzfeld source in the commercial SST-1 device, the mock-up of the dual diffuser stack in the Portable Field Dark Adaptometer, and the ideal Lambertian reflectance across an angular illumination range of -70 to +70°

As shown in Fig. 3, the dual diffuser stack produced a distribution that was slightly broader than the ideal Lambertian distribution (i.e., uniform scatter) and may represent a minor level of inherent measurement error or a mild, non-uniform illumination from the LED itself that was not completely dispersed by the dual diffuser stack.

Testing confirmed the functionality of the PFDA under challenging field conditions. Its size and weight allowed it to be carried long distances to areas unreachable by car. Minimally trained field workers collected readable videos in a variety of settings, often outside in bright sunlight. Testers encountered some early challenges in connecting the PFDA convertor box/goggles to the computer associated with the use of older “Apple talk” cables, which were replaced by standard USB cables. There were also issues with computer battery life, which were resolved in one field setting by switching from laptop to netbook computers. Examiners carried out an average of ~10 tests per day. Across all four populations, the device was acceptable to a range of subjects from pregnant women to young, preschool-aged children. No tests were terminated due to subject discomfort or concerns. In some cases, on exceptionally hot days, the headset resulted in a lot of perspiration around the eyes; this did not affect the test integrity. Subjects appreciated the disinfection of the goggles with disposable alcohol swabs between users.

A total of *n* = 822 videos were collected across the four sites, with mean (± SD) pupillary response ranging from -15.5 ± 5.8 log cd/m^2^ in Kenyan school children to −22.7 ± 8.0 log cd/m^2^ in Peruvian adults. Estimates of pupil dynamics were slowest in the Kenya dataset, similar to what was seen with pupillary response. In all studies, response time was faster to the low intensity stimuli; we also observed the greatest variance in response time between countries across the low intensity stimuli. Mean pupillary thresholds were within the same range as previously reported findings [7] and show a similar pattern as the other metrics; the highest threshold was in Kenya, where 33.7 % of children were classified as having impaired dark adaptation. Results were roughly equivalent in the data from Bangladesh and Peru, where the prevalence of impaired dark adaptation was ~10 %. Although biochemical data are not yet available to test this hypothesis, the marked variation in pupil response metrics across sites is likely due to differences in vitamin A status. Young children, in particular, are at an increased risk of vitamin A deficiency [19], which we suspect as the underlying cause of impaired dark adaptation in our preschool- and school-aged samples. Ethnic differences could conceivably influence our finding as well. However, the challenge of measuring pupil response in darkly pigmented eyes has largely been overcome with the use of infrared technology.

**Fig. 4:**
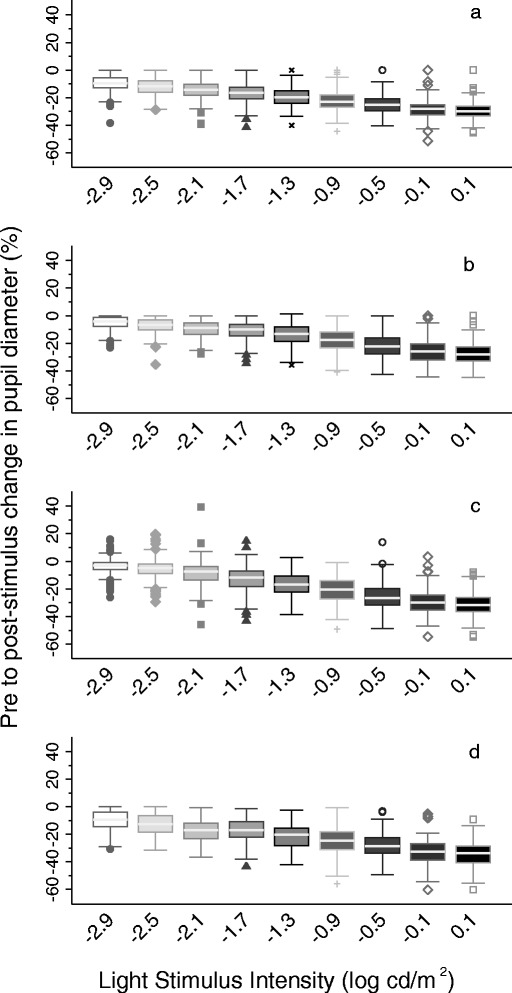
Trend in pupillary response across nine log-incremental steps of light intensity (left to right) in Bangladesh (**a**), Kenya (**b**), Zambia (**c**), and Peru (**d**). Pupillary response is defined as the percent change in pupil diameter from pre- to post-stimulus

There was a clear trend of increased pupillary response with increasing light intensity across all four sites (Fig. 4). Regression modeling confirmed a highly significant relationship between pupillary response and light intensity, irrespective of starting pupil diameter, with values ranging from −2.6 log cd/m^2^ (95 % CI: −2.7, −2.5) among reproductive-aged women in Bangladesh to −3.7 log cd/m^2^ (95 % CI: −3.9, −3.6) in Zambian preschool-aged children. While validation of the PFDA was beyond the scope of this work, the design of the PFDA was guided by the SST-1 device, which compares favorably to the Goldmann-Weekers Dark Adaptometer—previously considered the gold standard for assessing dark adaptation [13]. Employment of an infrared camera and objective video analysis may represent an advance over these previous psychophysical testing protocols. We expect that the PFDA’s automated test procedure would also improve the reliability of dark adaptation assessments by reducing variability associated with examiner technique [20]. We did note some instances, particularly in the Zambia dataset (2 % of measurements; 12 % of children), of pupil dilation in response to light stimuli (Fig. 4). While not statistically significant, the mean age of children with implausible values was lower than that of their peers (difference = 0.37 ± 0.44 years; *p* = 0.1). These implausible values likely reflect the challenge of working with younger subjects, who may be less able to keep their eyes fixed straight ahead for the entire testing period, thus complicating pupil diameter measurements. They also underscore the need for close supervision of examiners in the field, who must continuously monitor eye position on the computer screen and encourage their subjects, particularly children, to look straight ahead with their eyes wide open during the testing phase.

## Conclusions

Field testing of the PFDA confirmed its ease of use for measuring dark adaptation, even under challenging field conditions. Testing in four populations clearly replicated the well-recognized linear relationship between pupillary responsiveness over log-incremental increases in light intensity. We also noted faster response times at the lower light intensity stimuli, indicative of the slight pupillary fatigue experienced between the beginning of the test, after 10 min of dark adaptation, and the nine consecutive, log-incremental pupillary stimuli. The indices of pupillary response and pupil dynamics generated here from PFDA measurements are not exhaustive. However, we do recommend that future PFDA studies report pupillary threshold to ensure comparability with previous research [7], and in the case of vitamin A deficiency-related research, consider a similar focus on the high light intensity stimuli.

The PFDA was initially designed for use in public health nutrition research and practice. Based on the previous findings showing a direct relationship between pupillary responsiveness and serum retinol concentration [8, 14, 15] and modified dose response [8], the PFDA is currently undergoing validation as a tool to screen populations for vitamin A deficiency. It also being employed in efficacy trials of provitamin A carotenoid biofortified foods [21], where dark adaptation is a primary functional outcome. There are potential applications for the PFDA in research on non-communicable diseases and clinical practice as well. For example, it might serve as a rapid and non-invasive status test for patients with complex gastrointestinal and hepatobiliary conditions linked to poor vitamin A absorption [22]. Dark adaptometry can also be employed in the diagnosis of age-related macular degeneration, photoreceptor dystrophies, and glaucoma [23–25]. Finally, there is a well-established, causal relationship between poor scotopic vision and hypoxemia [3], which suggests a potential application of this device in the search for early predictors of cardiopulmonary disease.

## Additional files

Additional file 1:
**Example PFDA video from a child with normal dark adaptation.**
Additional file 2:
**Example PFDA video file from a child with abnormal dark adaptation.**

## Abbreviations

PFDA: Portable Field Dark Adaptometer
SST-1: Scotopic Sensitivity Tester-1

## References

[1] Koch W (1945). A new instrument for dark adaptation tests. Br J Ophthalmol.

[2] Working Group on Night Vision. Night Vision: Current Research and Future Directions, Symposium Proceedings. Washington, D.C.: National Academies Press; 1987.

[3] Havelius U, Hansen F, Hindfelt B, Krakau T (1999). Human ocular vasodynamic changes in light and darkness. Invest Ophthalmol Vis Sci.

[4] Havelius U, Bergqvist D, Falke P, Hindfelt B, Krakau T (1997). I. Impaired dark adaptation in symptomatic carotid artery disease. Neurology.

[5] Lamb TD, Pugh EN (2006). Phototransduction, dark adaptation, and rhodopsin regeneration the proctor lecture. Invest Ophthalmol Vis Sci.

[6] Carr CJ, Fisher KD (1970). A study of individual variability in dark adaptation and night vision in man.

[7] Congdon NG, West KP (2002). Physiologic indicators of vitamin A status. J Nutr.

[8] Congdon N, Sommer A, Severns M, Humphrey J, Friedman D, Clement L, et al. Pupillary and visual thresholds in young children as an index of population vitamin A status. Am J Clin Nutr. 1995;61(5):1076–82. 10.1093/ajcn/61.4.1076 7733032

[9] Dowling JE, Wald G (1958). Vitamin A Deficiency and Night Blindness. Proc Natl Acad Sci U S A.

[10] Wald G (1941). A portable visual adaptometer. J Opt Soc Am.

[11] Wilson WH (1946). The Middle East Adaptometer. Br J Ophthalmol.

[12] Yarbrough ME, Dann WJ (1941). Dark adaptometer and blood vitamin A measurements in a North Carolina Nutrition Survey. J Nutr.

[13] Peters AY, Locke KG, Birch DG (2000). Comparison of the Goldmann-Weekers dark adaptometer and LKC Technologies Scotopic Sensitivity tester-1. Doc Ophthalmol.

[14] Congdon NG, Dreyfuss ML, Christian P, Navitsky RC, Sanchez AM, Wu LS, et al. Responsiveness of dark-adaptation threshold to vitamin A and beta-carotene supplementation in pregnant and lactating women in Nepal. Am J Clin Nutr. 2000;72(4):1004–9. 10.1093/ajcn/72.4.1004 11010944

[15] Sanchez AM, Congdon NG, Sommer A, Rahmathullah L, Venkataswamy PG, Chandravathi PS, et al. Pupillary threshold as an index of population vitamin A status among children in India. Am J Clin Nutr. 1997;65(1):61–6. 10.1093/ajcn/65.1.61 8988914

[16] West Jr KP, Christian P, Labrique AB, Rashid M, Shamim AA, Klemm RD, et al. Effects of vitamin A or beta carotene supplementation on pregnancy-related mortality and infant mortality in rural Bangladesh: a cluster randomized trial. JAMA. 2011;305(19):1986–95. 10.1001/jama.2011.656 21586714

[17] World Health Organization (2009). Global prevalence of vitamin A deficiency in populations at risk 1995–2005. WHO Global Database on Vitamin A Deficiency.

[18] Miranda JJ, Bernabe-Ortiz A, Smeeth L, Gilman RH, Checkley W, Cronicas Cohort Study Group. Addressing geographical variation in the progression of non-communicable diseases in Peru: the CRONICAS cohort study protocol. BMJ Open. 2012;2(1), e000610. 10.1136/bmjopen-2011-000610 PMC3278488 22240652

[19] Sommer A, West Jr KP. Vitamin A and childhood morbidity. Lancet. 1992;339(8804):1302. 10.1016/0140-6736(92)91637-n 1349704

[20] Kiser AK, Mladenovich D, Eshraghi F, Bourdeau D, Dagnelie G (2006). Reliability and consistency of dark-adapted psychophysical measures in advanced eye disease. Invest Ophthalmol Vis Sci.

[21] De Moura FF, Palmer AC, Finkelstein JL, Haas JD, Murray-Kolb LE, Wenger MJ, et al. Are biofortified staple food crops improving vitamin a and iron status in women and children? New evidence from efficacy trials. Adv Nutr. 2014;5(5):568–70. 10.3945/an.114.006627 PMC4188236 25469399

[22] Abbott-Johnson WJ, Kerlin P, Abiad G, Clague AE, Cuneo RC (2011). Dark adaptation in vitamin A-deficient adults awaiting liver transplantation: improvement with intramuscular vitamin A treatment. Br J Ophthalmol.

[23] Jackson GR, Edwards JG (2008). A short-duration dark adaptation protocol for assessment of age-related maculopathy. J Ocul Biol Dis Inform.

[24] Kalaboukhova L, Fridhammar V, Lindblom B (2007). Relative afferent pupillary defect in glaucoma: a pupillometric study. Acta Ophthalmol Scand.

[25] Owsley C, McGwin G, Jackson GR, Kallies K, Clark M (2007). Cone- and rod-mediated dark adaptation impairment in age-related maculopathy. Ophthalmology.

